# Malaria care-seeking behaviour among HIV-infected patients receiving antiretroviral treatment in South-Eastern Nigeria: A cross-sectional study

**DOI:** 10.1371/journal.pone.0213742

**Published:** 2019-05-09

**Authors:** Uchechukwu M. Chukwuocha, Gregory N. Iwuoha, Geoffrey C. Nwakwuo, Peter K. Egbe, Chidinma D. Ezeihekaibe, Christopher P. Ekiyor, Ikechukwu N. S. Dozie, Sahai Burrowes

**Affiliations:** 1 Department of Public Health, Federal University of Technology, Owerri, Nigeria; 2 RAHI Medical Outreach, Port Harcourt, Nigeria; 3 College of Education and Health Sciences, Touro University, California, United States of America; Imperial College London, UNITED KINGDOM

## Abstract

This study assesses malaria prevention and treatment behaviour among people living with HIV/AIDS (PLWHA) in Owerri, South Eastern Nigeria. Although Nigeria bears one of the world’s largest burdens of both malaria and HIV, there is almost no research studying how co-infected patients manage their care. We systematically sampled 398 PLWHA receiving care at Imo State Specialist Hospital and the Federal Medical Centre in Owerri to complete a structured, pre-tested questionnaire on malaria care-seeking behaviour. Descriptive statistics were reported and chi-square tests and multivariate logistic regressions were also used. The majority of HIV-infected patients (78.9%) reported having had an episode of suspected malaria quarterly or more often. There was a large variation in care-seeking patterns: on suspicion of malaria, 29.1% of participants engaged in self-medication; 39.2% went to drug shops, and only 22.6% visited HIV/AIDS care centres. Almost 40% waited more than 24 hours before initiating treatment. Most (60.3%), reported taking recommended artemisinin-based combination treatments (ACT) but a significant minority took only paracetamol (25.6%) or herbal remedies (3.5%). Most (80%) finished their chosen course of treatment; and completion of treatment was significantly associated with the frequency of suspected malaria occurrence (p = 0.03). Most (62.8%) did not take anti-malaria medication while taking antiretroviral treatment (ART) and almost all (87.6%) reported taking an ACT regimen that could potentially interact with Nigeria’s first-line ART regimen. Our findings suggest the need to pay more attention to malaria prevention and control as a crucial element in HIV/AIDS management in this part of Nigeria and other areas where malaria and HIV/AIDS are co-endemic. Also, more research on ART-ACT interactions, better outreach to community-level drug shops and other private sector stakeholders, and clearer guidelines for clinicians and patients on preventing and managing co-infection may be needed. This will require improved collaboration between programmes for both diseases.

## Introduction

Malaria and HIV/AIDS overlap geographically in sub-Saharan Africa, Southeast Asia, and South America. Both diseases pose enormous global health challenges with each causing over three million deaths in 2007 and adversely affecting millions more each year [1, 2]. Nigeria has the world’s largest malaria burden with more than 50 million cases annually and approximately 200,000 malaria-related deaths each year [3]. It also has the world’s second largest HIV/AIDS burden, with approximately 3.6 million people living with HIV/AIDS in 2016 [4]. In Imo State, South Eastern Nigeria, an area reported to be holo-endemic for malaria [5], the HIV prevalence rate is now 7.5%, which is above the national average of 3.0% [6].

Co-infection with malaria and HIV can make each disease more severe and potentially more infectious. Studies have shown that malaria infection increases plasma HIV viral load even with asymptomatic parasitaemia [7–9]. In turn, the risk of parasitaemia and malarial fever increase with decreasing CD4 cell counts and increasing viral load [8, 10]. Although early studies found little association between HIV and malaria disease severity in either adults or children [11, 12], it has subsequently been demonstrated that HIV infection predisposes both to more frequent episodes of symptomatic malaria [13] and more episodes of severe or complicated malaria, including death [14]. HIV-malaria co-infection is particularly serious for pregnant women in whom complications such as life-threatening anaemia and placental malaria infection are common [15]. Individuals in malaria-endemic areas who are considered semi-immune to malaria are still more likely to develop clinical malaria if they are infected with HIV [16].

Co-morbidity also complicates treatment for both diseases as many standard, first-line treatments for malaria and for HIV interact; placing people living with HIV/AIDS (PLWHA) at high risk of harmful adverse events including hepatotoxicity if incorrect antiretroviral drug regimens are prescribed [17–19]. If patients stop antiretroviral HIV treatment while co-infected, this incomplete or interrupted treatment can lead to resistance, making further treatment more difficult and costlier. In addition, poorly managed HIV infection reduces responses to malaria treatment by weakening the immune system. For example, the effectiveness of artemisinin, sulfadoxine-pyrimethamine, and artemether-lumefantrine have been found to vary in PLWHA [20].

Considering the high rates of co-morbidity and the significant treatment challenges posed by malaria and HIV co-infection, it is important to understand how PLWHA are currently preventing and treating malaria. However, while there is an extensive literature on malaria care-seeking behaviour in Africa [19–23] there are almost no studies of the malaria care-seeking behaviour of PLWHA in general, and those receiving antiretroviral treatment (ART) in particular. As a result, we know little about the care-seeking behaviour and patterns of malaria prevention and treatment among PLWHA. We might suspect that the behaviour of PLWHA might differ from that of the general population given their intense contact with the health system and the relatively high level of resources available in HIV-treatment facilities.

This study therefore aims to describe malaria care-seeking behaviours among HIV-infected patients receiving antiretroviral treatment in South-Eastern Nigeria. Our specific research questions are: 1) How common is self-reported malaria co-infection among this sample of PLWHA? 2) To what extent do PLWHA in our sample use informal services (including self-treatment) to diagnose and treat suspected malaria? 3) What medications do PLWHA in our sample use to treat suspected malaria? 4) Do PLWHA alter their antiretroviral treatment practices when taking anti-malarial drugs?

It is hoped that the study results will provide information to inform the development and application of effective interventions that would improve the prevention and management of malaria among HIV-infected patients.

## Materials and methods

### Study location

The study was carried out in Owerri, South Eastern Nigeria, at the Imo State Specialist Hospital (IMSSH) and the Heart-to-Heart unit of the Federal Medical Centre (FMC). The HIV care centres in these facilities are among the major sources of comprehensive HIV treatment and support for PLWHA in South Eastern Nigeria. South-Eastern Nigeria is one of the geopolitical zones in Nigeria, consisting of five states and inhabited by the Igbo-speaking ethnic group. The area covers a landmass of 41,440 km², characterized by tropical rainforest vegetation. Apart from state capitals and a few other urban towns, the area is rural. South-Eastern Nigeria has two distinct seasons, the rainy and the dry seasons. Its climate and environment provide favourable conditions for the breeding of the mosquito vector and malaria endemicity. The area has many socioeconomic characteristics that increase the population-level risk of HIV infections. Malaria co-infection among asymptomatic and symptomatic HIV seropositive individuals in South-Eastern Nigeria has been reported to be 11.8% and 33.3% respectively [24].

### Study design

The study has a descriptive, cross-sectional design. The study population comprised of all patients living with HIV registered in Imo State Specialist Hospital in Owerri and Federal Medical Centre in Owerri, from October 1 to December 31, 2017, as noted in hospital records (N = 2,300). The study sample size was determined using the Taro Yamane method for survey-related studies [25], to obtain a minimum sample size of 341.

$$n=\frac{N}{1+N\left(e^{2}\right)}$$ (1)

Because of HIV-related stigma and the tendency for PLWHA to shy away from public activities, we envisaged that a significant proportion of HIV-infected patients would decline to participate in the study. We, therefore, oversampled an additional 20% of the calculated sample size to account for attrition and arrived at a sample size of 409. However, nine of the selected participants did not give consent to participate in the study, leaving us with the final sample size of 398.

### Sampling technique

Systematic sampling was used to select study subjects from both study HIV care centres. We sampled patients over four weeks (two weeks for each centre) during the clinic appointment days for PLWHA. Initially, the number of eligible participants was obtained from the records at each HIV care centre. Only those who were available on the appointment days were selected. On each appointment day, we first divided the number of expected patients who had appointments as listed on the health centre’s records, by the required sample size to obtain an interval number. This interval number was then used to systematically sample patients as they arrived for their appointments on their respective appointment days. For example, if the day’s interval was five, each fifth patient who arrived for an appointment was recruited to participate in the study. In situations when a selected patient declined to consent to participate, the next patient to arrive was selected. The records were crosschecked to ensure that subjects were not selected more than once.

### Development and pre-testing of study instrument

The instrument for data collection was a structured pre-tested questionnaire (see S1 Appendix). The questionnaire was developed by the study researchers in collaboration with three HIV experts. The questionnaire was pre-tested using 30 HIV-infected patients at another, non-study, HIV care centre in Imo state. The questionnaire contained 37 open- and closed-ended questions on the respondents’ demographic background; HIV treatment history; and malaria care-seeking behaviour; including the frequency of malaria episodes; type, source and timing of malaria medication or diagnostics used; and whether the patient took anti-malaria drugs while receiving antiretroviral therapy (see S1 Appendix). The survey was created in English and translated into Igbo and Pidgin English by the study researchers. Pre-test data were not included as part of the present study. Adjustments were made after pre-testing, before the questionnaire was used for study data collection.

### Data collection

Data were collected using the structured, pre-tested questionnaire by a team of trained research assistants under the supervision of study investigators. To each study participant, the study was first introduced, and the objectives elucidated. Informed verbal consent was also sought and obtained before the questionnaire was administered. Literate respondents self-administered the survey in either English, Igbo, or “Pidgin English”. Research assistants stayed on hand to clarify points of confusion and collected the questionnaires immediately after completion. For patients who could not self-administer the survey either due to illness or lack of literacy, the research assistants verbally administered the questionnaire to them in the local (Igbo) language, English or “Pidgin English” based on the subjects’ choice. The responses were then immediately translated and/or transcribed into English and filled out on the questionnaire by the trained research assistant. Data collection occurred over a one-month period (see S1 Table).

### Data analysis

Initial analysis consisted of calculating descriptive statistics to create percentage and frequency tables. Pearson’s chi-squared test (χ2) was performed to examine associations between completing anti-malarial treatments, patterns of reported suspected malaria (re)-occurrence, and having adverse reactions to simultaneous ART and malaria treatment. We also performed multivariate logistic regression to assess associations between socio-demographic factors such as educational attainment, urban residence, and gender and the use of formal or recommended healthcare services for malaria diagnosis and treatment. Probability values (*p*) less than or equal to 5% were considered statistically significant. Analyses were performed on IBM-SPSS Statistics version 23 and Stata version 14.

### Variables of interest

Respondents were said to have used “formal” services if they received treatment at licensed pharmacies, general clinics or hospitals, or HIV care centres. Using microscopy, rapid tests, or a clinical exam for diagnosis was considered receiving a formal diagnosis and getting a medication prescription from a clinician rather than self-medicating or purchasing medication from drug-shops was considered using formal prescription services. Respondents who answered, “self-treatment at home” to the question of “What action do you take when you suspect malaria?” were considered to be self-treating. Patients were asked to name the drugs that they used for treating malaria. If they selected an artemisinin-based compound combined with a drug from a different class, they were coded as using ACTs. Respondents were asked directly whether they took antiretrovirals and anti-malarial simultaneously and which kind of adverse event (e.g., nausea, rash) related to their treatment they had experienced. If they selected any of the adverse events from the list, they were considered as having had an adverse event. These six measures were coded as dichotomous outcome variables for our multivariate logistic regression analysis of factors associated with healthcare seeking behaviour.

### Ethical approval

Ethical approval was given by the Ethical Committee of the School of Health Technology, Federal University of Technology Owerri, Nigeria. Permission was also sought and obtained from the management of Imo State Specialist Hospital Owerri and the Federal Medical Centre Owerri, South Eastern Nigeria. Informed verbal consent as permitted by the Ethical Committee was sought and obtained from all the participants before they could take part in the study. Research assistants read informed consent form material to participants and noted the respondents’ consent on the form when given. Verbal consent was obtained instead of written consent because of concerns about patient literacy and protecting patient confidentiality considering the heavy stigma that HIV-infection can carry in this region.

## Results

### Socio-demographic characteristics

Table 1 shows the socio-demographic characteristics of the sample. A total of 398 PLWHA participated: 210 (53%) from Federal Medical Centre (FMC) and 188 (47%) from Imo State Specialist Hospital (IMSSH). Our response rate was 97.3%. There were slightly more women than men in the study sample: 221 males (46%) and 259 females (54%). More than half of the subjects (55%) were married, 36% were single, 7.5% divorced, and 1.5% widowed. The sample was relatively well educated, with 57% having achieved a secondary school education or higher. The relatively high socio-economic status is reflected in reported income, with more than half of the sample reporting income well above Nigeria’s poverty line. The majority were employed in the public sector or in trading or small businesses (see Table 1).

**Table 1:** Socio-demographic characteristics.

Characteristics	Frequency (n = 398)	Percent
**Location**		
FMC OW	210	52.8
IMSSP	188	47.2
**Gender**		
Male	160	40.2
Female	238	59.8
**Age (in years)**		
≤ 25	102	25.6
26–35	176	44.2
36–45	78	19.6
≥46	32	8.0
Non-response	10	2.5
**Marital status**		
Single	144	36.2
Married	218	54.8
Divorced	30	7.5
Widowed	6	1.5
**Education**		
Tertiary	118	29.6
Secondary	190	47.7
Primary	68	17.1
Non-formal	22	5.5
**Occupation**		
Student	140	35.2
Business	194	48.7
Public servants	48	12.1
Artisans	10	2.5
Others	4	1.0
Non-response	2	0.5
**Monthly income (naira)**		
≤ 18,000	184	46.2
19,000–60,000	208	52.3
61,000–100,000	6	1.5
≥ 100,000	0	0.0
**Duration lived with HIV**		
< 1 year	44	11.1
1–5 years	192	48.2
6–10 years	152	38.2
>10 years	10	2.5
**Receiving antiretroviral therapy**		
Yes	390	98.0
No	8	2.0
**Knowledge of the drugs used for their therapy**		
Yes	156	39.2
No	238	59.8
Non-response	4	1.0

### HIV status

There were very few newly HIV-diagnosed patients; only 11% had lived with HIV for less than a year; 48% had been aware of their status for 1–5 years and 38% for 6–10 years (see Table 1). Almost all (98%) were currently taking ART but only 39% of patients receiving ART knew which drugs were used for their therapy.

### Patterns of self-malaria occurrence

Table 2 reports the extent of malaria occurrence reported among the HIV/AIDS patients studied. All study subjects said they had experienced malaria while receiving HIV/AIDS care. More than one-third (36%) of HIV-infected patients had a reported malaria occurrence every month and 43% and 16% respectively reported experiencing malaria on a quarterly and yearly basis.

**Table 2:** Self-reported experiences of suspected malaria.

	Frequency (n = 398)	Percent
**Have ever had malaria (suspected)**		
Yes	398	100
No	0	0.0
**Frequency of suspected malaria occurrence**		
Monthly	142	35.7
Quarterly	172	43.2
Yearly	64	16.1
Other	20	5.0

### Patient reaction to suspicion of malaria

Few patients sought appropriate, formal care when they suspected that they had malaria. The plurality (39%) used drug shops to buy malaria drugs from shop attendants when they suspected an attack and most others (29%) self-treated at home. Only 23% reported their suspicion to their HIV/AIDS care centres for proper diagnosis (see Table 3). Most respondents (57%) said that they acted within 24 hours of a suspected malaria occurrence.

**Table 3:** Reaction on suspicion of malaria.

Reaction/ Behaviour	Frequency (n = 398)	Percent
**Action taken on suspicion of malaria attack**		
Self-treatment at home	116	29.1
Go to drug-shop	156	39.2
Report to HIV/AIDS care centre	90	22.6
Go for medical laboratory diagnosis	34	8.5
Others	2	0.5
**Time taken to commence treatment of suspected malaria**		
≤ 24 hours	226	56.8
≥ 24 hours	150	37.7
Can’t remember	20	5.5
**Usual method of confirmation of suspected malaria**		
Microscopy test	108	27.1
Rapid diagnostic test	64	16.1
Doctors clinical examination	168	42.2
No confirmation	48	12.1
Other	4	1.0
Non-response	6	1.5
**Anti-malaria drugs prescriber**		
Self	214	53.8
Drug shop owners	106	26.6
Doctor	64	16.1
Friend/relative	14	3.5

When seeking formal confirmation for suspected malaria an almost equal proportion rely on clinical examination (42%) or laboratory tests (27% microscopy, 16% rapid diagnostic tests). Approximately 12% do not use any form of formal confirmation of diagnosis.

Self-treatment was less prevalent among respondents with no formal schooling (AOR = 0.21; 95% CI = 0.05,0.95) than among those who had completed primary, secondary, or tertiary schooling. There was also some indication that men in this sample were less likely to self-treat than women although this association was only significant at the 10% level (AOR = 0.66; 95% CI = 0.41,1.05).

### Malaria treatment drugs: Sources, types and usage patterns

Only a small minority of patients received their malaria treatment medications from the formal sector (see Table 4). More than half (54%) of study subjects said that their primary source of anti-malaria drugs was drug shops. Slightly less than a third (28%) used licensed pharmacies to purchase drugs, and only 16% received medications from clinics/hospitals. HIV clinics were rarely used as a source of medication. We found no significant differences in the use of formal vs. informal sources for purchasing malaria treatment medications by gender, educational level or urban residence (See Table 5).

**Table 4:** Malaria treatment medication: Sources, types and usage patterns.

Assessment	Frequency (n = 398)	Percent (%)
**Source of anti-malaria drug**		
Drug shop	216	54.3
Licensed pharmacy	110	27.6
Clinic/hospital	64	16.1
HIV/AIDS care centre	8	2
**Primary drug used for malaria treatment**		
ACT	240	60.3
Antipyretics (paracetamol)	102	25.6
Other anti-malaria drugs (not ACT)	38	9.5
Herbs	14	3.5
Other treatments	4	1
**ACT type used if ACT (n = 240)**		
Arthemeter/lumefantrine	144	60.0
Artesunate/amodiaquine	66	27.6
Dihydroartemisinin/piperaquinet	26	10.8
Arthesunate + mefloquine	2	0.8
Chloroguanil+dapsone+artesunate	2	0.8
**Completion of malaria treatment**		
Yes	320	80.4
No	78	19.6
**Takes anti-malaria drug & ART at the same time**		
Yes	148	37.2
No	250	62.8

**Table 5:** Logistic regression: Factors associated with healthcare-seeking behaviour.

Variable	Variable class	Receives Drugs from Formal Sector		Uses Formal Malaria Confirmation Methods		Self-Treatment for Malaria		Uses ACT for Malaria Treatment		Takes Antimalarials with ART		Experiences Adverse Reaction with ART &Antimalarials	
	n (%)	AOR	95% CI	AOR	95% CI	AOR	95% CI	AOR	95% CI	AOR	95% CI	AOR	95% CI
**Gender**													
Female	238 (59.8)	Base		Base		Base		Base		Base		Base	
Male	160 (40.2)	1.14	[0.746,1.725]	1.61	[0.836,3.116]	0.66	[0.410,1.049]	1.27	[0.824,1.963]	1.37	[0.864,2.182]	1.45	[0.813,2.592]
**Age(in years)**													
25 or younger	102 (25.6)	Base		Base		Base		Base		Base		Base	
26–35	176 (44.2)	1.51	[0.912,2.494]	1.03	[0.451,2.359]	0.97	[0.554,1.692]	2.11**	[1.266,3.522]	1.36	[0.789,2.340]	4.17***	[1.783,9.769]
36–45	78 (19.6)	1.38	[0.746,2.547]	0.61	[0.248,1.518]	1.32	[0.686,2.555]	1.51	[0.811,2.796]	0.58	[0.282,1.189]	3.66**	[1.370,9.754]
46 or older	32 (8.0)	1.20	[0.530,2.699]	0.28*	[0.101,0.780]	1.05	[0.435,2.524]	2.77*	[1.165,6.592]	1.34	[0.537,3.316]	2.44	[0.686,8.675]
**Residence**													
Rural	184 (46.2)	Base		Base		Base		Base		Base		Base	
Urban Residence	214 (53.8)	0.95	[0.630,1.425]	0.43**	[0.229,0.799]	1.11	[0.711,1.740]	0.75	[0.491,1.139]	0.80	[0.507,1.266]	3.69***	[2.045,6.644]
**Educational Level**													
No Formal Education	22 (5.5)	0.66	[0.262,1.672]	1.36	[0.285,6.430]	0.21*	[0.048,0.952]	0.68	[0.274,1.698]	3.77*	[1.356,10.46]	0.29	[0.0569,1.449]
Primary	68 (17.1)	1.02	[0.573,1.797]	0.85	[0.375,1.937]	1.27	[0.695,2.310]	1.21	[0.673,2.161]	1.29	[0.687,2.440]	0.21**	[0.0708,0.640]
Secondary	190 (47.7)	Base		Base		Base		Base		Base		Base	
Tertiary	118 (29.6)	1.17	[0.731,1.878]	1.04	[0.503,2.151]	0.86	[0.510,1.449]	1.82*	[1.108,2.997]	1.67	[0.995,2.798]	0.98	[0.532,1.800]
**Antimalarial treatment**													
ACT	240 (60.3)									Base		Base	
Antipyretics	102 (25.6)									1.18	[0.689,2.008]	1.15	[0.586,2.263]
Non-ACT antimalarial	38 (9.5)									3.94***	[1.875,8.257]	1.85	[0.755,4.535]
Herbs	14 (3.5)									6.14**	[1.729,21.80]	1.97	[0.487,7.971]
Other treatments										omitted		omitted	
**Observations**		384		384		384		384		374		374	

* p < 0.05,

** p < 0.01,

*** p < 0.001

Most patients reported using recommended malaria treatment medications, with 60% saying that they used ACTs for treatment. However, a significant minority (26%) only used antipyretics (usually paracetamol) and other almost 10% used a non-ACT anti-malaria medication. Use of traditional medicines was low, with only 3.5% saying that they used herbs. The most common ACT regimens used were artemether/lumefantrine (60%) which is the treatment recommended by the Nigerian Ministry of Health, [26] artesunate/amodiaquine (28%), and dihydroartemisinin/piperaquine (11%). Respondents with tertiary education were more likely to use ACTs for malaria treatment than those with no formal schooling [AOR = 1.82; 95% CI: 1.11, 3.00]. Older respondents were significantly more likely to use ACT than the youngest age group: AOR = 2.11 (95% CI: 1.27, 3.52) for the 26–35 years age group, and AOR = 2.77 for the oldest age group, (95% CI: 1.17, 6.59) (See Table 5).

Approximately 80% of patients said that they usually complete their chosen malaria treatment course. Those who reported very frequent (monthly) or very infrequent (annual) suspected malaria occurrences were significantly more likely to complete their malaria treatments (p = 0.03) than those who reported quarterly occurrences (see Table 6). We found no significant socio-demographic differences in reported completion by type of malaria treatment regimen (See Table 5).

**Table 6:** Self-reported suspected malaria occurrence pattern and completion of treatment.

	Self-Reported Suspected Malaria Frequency				
	Monthly	Quarterly	Yearly	Other	p-value*
	n = 142	n = 172	n = 64	n = 20	
Complete treatment?					
Yes	112 (78.9%)	148 (86.0%)	44 (68.8%)	16 (80.0%)	0.03
No	30 (21.1%)	24 (14.0%)	20 (31.2%)	4 (20.0%)	

*Chi-squared test. “Other” includes those who reported having malaria but who were not sure of how often they experienced it.

We found that 62.8% of respondents said that they did not take their chosen anti-malaria drugs while they were taking their antiretroviral drugs. Respondents who took herbs or alternative drugs had significantly higher odds of continuing ART while taking malaria treatment than those taking ACTs. The adjusted odds of not stopping ART were 6.14 (95% CI: 1.73, 21.80) for patients who took herbs to treat their malaria and 3.94 (95% CI: 1.88, 8.26) for patients who took non-ACT malaria treatments (See Table 5). Respondents who took artesunate/amodiaquine for treatment had lower odds of continuing ART than those on other regimens (AOR = 0.53; 95% CI: 0.31, 0.91).

### Self-reported suspected malaria occurrence patterns and adverse events

Approximately one-fifth of respondents experienced adverse events related to malaria and HIV treatment. We performed a chi-square test to examine the relationship between these events and the frequency of self-reported suspected malaria, and found that the percentage of participants reporting adverse events differed significantly by self-reported malaria occurrence pattern (p<0.0001; χ^2^ = 24.5) with 22.5% of those who self-report having monthly bouts of suspected malaria reporting adverse events, 14.0% of those with reported quarterly attacks, and 18.8% of those reporting annual attacks respectively (see Table 7).

**Table 7:** Self-reported suspected malaria occurrence pattern and adverse events.

	Self-Reported Suspected Malaria Frequency				
	Monthly	Quarterly	Yearly	Other	p-value*
	n = 142	n = 172	n = 64	n = 20	
Adverse event					
Yes	32 (22.5%)	24 (14.0%)	12(18.8%)	12 (60.0%)	<0.0001
No	110(77.5%)	148(86.0%)	52 (81.2%)	8(40.0%)	

*Chi-squared test. “Other” includes those who reported having malaria but who were not sure of how often they experienced it.

Urban respondents (AOR = 3.69, 95%CI: 2.05, 6.44) were more likely to report adverse events than rural and respondents in the middle age groups than the youngest age group (AOR = 4.17 95%CI: 1.783, 9.769 for the 26–35 age group and AOR = 3.66, 95%CI: 1.370,9.754 for the 36–45 age group) (see Table 5). Those with less education had a lower likelihood of reporting adverse events (AOR = 0.213, 95%CI: 0.07, 0.64 for those with primary education compared to those with secondary education) (see Table 5).

### Patient recommendations for improving management of malaria/HIV co-infection

Almost none of the respondents (87%) could provide a recommendation for ways to improve malaria prevention and treatment for PLWHA. The few suggested actions that were offered were combining malaria treatment with HIV drugs (1%), suspending antiretroviral drugs while on malaria treatment (7%), and using treated mosquito nets to prevent further mosquito bites (4%) (see Table 8).

**Table 8:** Patient recommendations for improving management of malaria/HIV Co-infection.

Recommendations	Frequency (n = 398)	Percent (%)
Combine malaria treatment with HIV Drugs	4	1.0
Suspend use of HIV drugs while on malaria treatment	28	7.0
Use of treated mosquito net	16	4.0
Constant testing against malaria	2	0.5
Don’t Know	348	87.4
Total	398	100

## Discussion

This study examined malaria care-seeking behaviour among HIV/AIDS patients receiving antiretroviral therapy in Southeastern Nigeria. We found extremely high levels of self-reported co-infection with all patients reporting having had suspected malaria and almost 80% reporting a suspected re-occurrence on at least a quarterly basis. Our data suggest that most of these suspected cases are never formally diagnosed but rather assumed to be malaria and treated as such. This highlights the need for greater access to rapid diagnostic tests, which were only used by 16.1% of our sample.

The rate of malaria co-infection reported by our study participants is higher than that found in other sub-Saharan African settings [27], which is likely a reflection of the self-reported nature of our study data. We simply asked participants if they had suffered from malaria and how often, rather than conducting parasitological diagnosis of malaria infection. The population in this area of Nigeria is exposed to a host of infectious agents that might cause malarial symptoms that participants may have mistakenly attributed to malaria. Our estimate of malaria occurrence in this population is, therefore, quite imprecise and most likely an overestimate.

We found heavy use of informal sources such as drug shop sellers and home-care for immediate treatment on suspicion of malaria. Self-medication and use of informal drug sellers are well documented among the general population in Nigeria [28, 29] and sub-Saharan Africa [30–32] and the rates reported here are in keeping with this literature. For example, this pattern of care is consistent with results from a study among HIV-infected pregnant women in Nigeria [33] that showed that half of the women self-prescribed anti-malaria drugs. The high cost of formal care, desensitization due to the high prevalence of malaria, shortages of drugs, and perceived low-quality care at formal facilities have been found as explanations for this phenomenon [31, 34].

What is surprising is that this study sample, which has steady access to relatively well-resourced health facilities, has treatment patterns like those found in the general public. This highlights the importance of drug cost (unlike antiretroviral drugs, malaria medications are not provided free of charge at HIV clinics in Nigeria), and of convenience factors in driving healthcare-seeking behaviour. The finding that self-treatment was more common among respondents in our study with higher levels of education lends additional support to the argument that convenience and quality of care are important drivers of self-treatment.

Our findings regarding the heavy use of drug sellers and private laboratories also underscore the continued importance of engaging the private sector in HIV/AIDS and malaria program development and of pursuing community-based treatment strategies to improve access to appropriate treatment. Our study and others indicate that there is a broad range of healthcare stakeholders in both the public and private sectors that are involved in malaria control among HIV-infected patients, including hospital clinics, pharmacies, private clinics, and patent medicine stores. For example, a recent Nigerian study reported that the private sector took care of more than 43% of malaria cases [35].

We find that the majority of patients using anti-malaria medications (as opposed to drugs for symptom relief) reported using the first-line, recommended ACTs. There were few reports of mono-therapy or of ineffective treatment, and approximately 80% of patients said that they finished their course of treatment. Although the quality of these medications is unknown as many were purchased privately, it is still heartening to find that newer, recommended treatment regimens are widely available and being taken. We must note, however, that a significant minority of respondents took only paracetamol, sub-optimal anti-malarial treatments, or herbal remedies.

We note two areas of concern regarding HIV and malaria co-treatment. First, many patients report using ACT regimens containing drugs (e.g., amodiaquine) that interact with the antiretroviral drugs included in Nigeria’s first line ART regime (namely, efavirenz). A significant minority of study patients combine anti-malaria drugs with ART, suggesting a risk for adverse events. In addition, patients were more likely to continue ART when they were taking non-ACT anti-malaria medication or herbal remedies (which may explain why less educated patients were more likely to report continuing ART than their more educated counterparts).The association between continuing ART and using traditional or non-ACT medications may be due to patients experiencing fewer drug interactions when taking these alternative medications or to them perceiving alternative regimens as relatively safer or milder and, therefore, being less afraid of remaining on ART while taking them. Other studies in sub-Saharan Africa have found that HIV-infected patients do concurrently use herbal treatment while on ART for perceived minor ailments (including malaria) in the belief that it increases their energy and immunity [36]. Although the number of people using traditional medication to treat malaria is low in our sample (which is in keeping with the figures reported in other studies of patients on ART [36]), our findings suggest that there may be potential, under-appreciated risk of interaction between traditional medication and ART in this population. The use of traditional medicines in patients who are taking ART and/or anti-malarial warrants further study, preferably using in-depth, qualitative methods that can better illuminate their beliefs and their decision-making processes than quantitative surveys such as ours.

Another concerning finding was that 80% of patients stopped taking ART when they started malaria treatment—a behaviour that increases the risk of resistance to antiretroviral medications and leads to poor viral load suppression. Moreover, the responses (or lack thereof) to the question asking for suggestions about to improve care, suggest that patients did not know the proper course of action for managing co-infection. Given the complexities of malaria treatment in patients receiving ART and the extremely high self-reported malaria incidence, more effort must be made to strengthen and develop new malaria prevention interventions and to better integrate them into HIV/AIDS care. Effective prevention interventions exist. For example, we know that co-infection is significantly lower among patients who use insecticide-treated bed nets and co-trimoxazole chemotherapy [9, 37]. Even if a significant proportion of HIV-infected patients in this study were not truly co-infected, mistaking a fever for malaria, if they self-medicate or adjust their HIV treatment as though they were co-infected, their health could be compromised.

A surprising finding is that most of the subjects studied knew the names of the drugs they use to treat malaria. This contrasts with findings in a recent Nigerian study [33], in which many of co-infected pregnant women stated that they never had any knowledge of intermittent preventive treatment. This difference may be due to the relatively well-educated sample and their tendency to self-treat, which requires that they know the names of their medication.

The finding that almost no study respondents could give suggestions for managing or preventing co-infection suggests that the structure of counselling sessions may not provide enough time to discuss such topics and provide updates. More comprehensive counselling sessions using techniques such as question and answer sessions and video recordings might help to fill gaps in patient knowledge. Providers and HIV clinic management staff should also develop systems to more closely track which malaria drugs are being used by patients.

## Limitations

This study is limited by its narrow geographic scope and modest sample size and non-random sample selection strategy, which reduces its generalizability. The study is subject to both recall and social desirability bias as respondents may not remember past malaria episodes or treatment behaviour accurately and may report the health-seeking behaviour that they perceive as desirable. Problems with recall may be exacerbated by the fact that patients with a serious, life-threatening illness may be under great physical and emotional stress, which may impair memory. We attempted to address recall bias by having data collectors on-hand to reword or repeat questions, and prompt memory but this, in turn, may have introduced measurement error if questions were explained differently by different data collectors. The lack of back-translation of our survey instrument may have also introduced measurement error. Social desirability bias, if it occurred would have most likely lead to an under-reporting of the significant levels of informal care reported by participants. Finally, as noted at the start of this discussion, our estimates of malaria occurrence are based on self-report rather than diagnosis and, therefore, not precise. While this limits our ability to measure the level of co-infection in this population it does not greatly limit our ability to describe the care-seeking behaviour of these HIV-infected patients when they suspected that they have malaria.

## Conclusion

This study has shown that PLWHA receiving ART in our sample self-report extremely high levels of suspected malaria, and that very little of their subsequent malaria care occurs in the formal sector. More worryingly, frequent bouts of suspected malaria seem to lead to interruptions in ART as most patients do not take ART and anti-malaria drugs simultaneously. Patients who do continue ART while treating malaria often use alternative, non-ACT anti-malaria drugs or herbal remedies, neither of which would tend to have well documented drug-drug interaction profiles with antiretroviral drugs. Our findings suggest the need for more research on ART-ACT interactions; better outreach to community-level drug shops and other private-sector stakeholders; and clearer guidelines for clinicians and patients on monitoring and managing co-infection. Wider access to rapid malaria diagnostic tests might also reduce the high level of presumptive malaria self-treatment that we find. Providing anti-malaria drugs for free in HIV care programs may also be worth exploring. Overall, we suggest that malaria prevention and control be viewed as an important element in the treatment HIV/AIDS patients in malaria-endemic countries such as Nigeria. This will require improved collaboration between programs for both diseases.

## Supporting information

S1 Appendix Questionnaire_Assessment of malaria treatment behaviour among people living with HIV in Owerri Metropolis, Nigeria, 2017. (DOCX)

S1 Table Dataset_Assessment of malaria treatment behaviour among people living with HIV in Owerri Metropolis, Nigeria, 2017. (XLSX)
