# Supplementary material for: Malaria care-seeking behaviour among HIV-infected patients receiving antiretroviral treatment in South-Eastern Nigeria: A cross-sectional study
Source: PLoS One. 2019 May 9;14(5):e0213742. doi: 10.1371/journal.pone.0213742 (PMC6508638; doi:10.1371/journal.pone.0213742)
Supplement: S1 Appendix — (DOCX) [file pone.0213742.s001.docx]

**QUESTIONNAIRE**

**ASSESSMENT OF MALARIA TREATMENT BEHAVIOUR AMONG PEOPLE LIVING WITH HIV IN OWERRI METROPOLIS (2017)**

All information given in this research will be treated with utmost confidentiality and secrecy. Please answer truthfully to enable me achieve objective results.

Please tick as appropriate

**Section A: Characteristics of respondents**

***Instruction: Please tick (√) as appropriate***

1. Gender:
   1. Male ( )
   2. Female ( )
2. Age:
   1. ≤ 25 ( ),
   2. 26 – 35 ( ),
   3. 36 – 45 ( ),
   4. ≥46 ( ) specify_______
3. Marital Status:
   1. a. Single ( )
   2. b. Married ( )
   3. c. Divorced ( )
   4. d. Widowed ( )
4. Educational Attainment
5. Tertiary ( )
6. Secondary ( )
7. Primary ( )
8. No formal Education ( )
9. Occupation
10. Student ( )
11. Business ( )
12. Public servant ( )
13. Artisan ( )
14. Others (specify) _____________
15. Monthly Income
16. ≤ 18,000 naira ( )
17. 19,000- 60,000 naira ( )
18. 61,000- 100000 naira ( )
19. ≥ 100,000 naira ( )
20. Where do you reside?
    1. Urban ( )
    2. Rural( )

**SECTION B: HIV STATUS TREATMENT AND COUNSELLING**

1. Are you a confirmed HIVpatient?
2. Yes ( ) b. No( )
3. How long have you lived with HIV ……………..
4. ≤ 1year b. 1-5years c. 5-10years d. ≥ 10years
5. How do you feel about your diagnosis?
6. Positive ( )
7. Angry( )
8. Scared ( )
9. Suicidal ( )
10. Depressed ( )
11. Are you on antiretroviral therapy?
12. Yes ( ) b. No( )
13. Do you know the drugs for your therapy?
14. Yes( ) b. No( )
15. If yes, what is the name of the drug?…………………………………….
16. Where do you receive antiretroviral therapy?......................................
17. Do you undergo HIV / AIDS counseling?
18. Yes( ) b. No( )
19. If yes, Where do you receive counseling?...................................................
20. How often do you go for counseling?................................
21. Monthly b.Quarterly c. Every 2 months d. Other
22. Do you ever miss counseling ?
23. Yes( ) b. No ( )
24. How often? …………………….
25. What is your reason for missing counseling ?.....................................

**SECTION C: MALARIA TREATMENT BEHAVIOR**

1. Have you experienced malaria ?
2. Yes( ) b. No ( )
3. How often do you have malaria?
4. Monthly( ) b. Quarterly( ) c. Yearly( ) d. Other( )
5. How do you feel to suspect malaria?
6. Fever( )
7. Fever+headache ( )
8. Fever+chills ( )
9. Fever+headache+chills ( )
10. Others( )
11. What action do you take when you suspect malaria?
12. Self treatment at home ( )
13. Go to drug shop/chemist ( )
14. Go to HIV/AIDS care centre ( )
15. Go to medical laboratory ( )
16. herbal ( )
17. fOthers_______________________
18. How long does it take you to take action to treat suspected malaria?
19. ≤ 24 hours ( ) b. ≥ 24 hours ( ) c. cant’t remember
20. How do you confirm your suspected malaria before treatment?
21. Microscopy ( ) b. RDT ( ) c. Doctors clinical examination d. No confirmation ( ) d. Others ( )
22. If no confirmation before treatment why?_________________________
23. Who prescribes antimalarial drug for your malaria treatment?
24. Self ( ) b. Drug shop owner ( ) c. Doctor ( ) d. Friend/Relative ( ) e. Other ( )
25. What is your source of antimalarial drug?

a. Drug Shop( )

b. Licensed Pharmacy ( )

c. Clinic hospital ( )

d. HIV/AIDs care center ( )

e. Left over drugs ( )

f. Others ( )

1. What drugs do you use for malaria treatment ?

a. ACT( ) ( ) b. Antipyretics (paractamol) ( ) c. other antimalarials (not ACT) ( )d. Herbs( ) e. other treatment ( )

1. What informs your choice of treatment? ___________________________
2. If you use ACTs , which ACTs do you use?
3. Arthemeterlumefantrine( )
4. Artesunateamodiaquine( )
5. dihydroartemisininpiperaquine( )
6. arthesunate + mefloquine( )
7. chloroguanil + dapsone + artesunate( )
8. Do you complete your antimalarial dosage?
9. Yes ( ) b. No ( )
10. Do you take antimalarials at the same time with antiretrovirals?
11. Yes ( ) b. No ( )
12. What adverse reactions do you experience?
13. Fever ( ) b. Nausea ( ) c. Body aches ( ) d. Vomiting ( ) e .rashes ( ) f. others __________________
14. Do you experience any difficulty in treating /managing malaria ?___________
15. Which difficulties do you experience in treating/managing malaria? _______
16. How do you think malaria treatment can be improved for PLWHA?

______________________________________________________
